# Lactational Amenorrhea: Neuroendocrine Pathways Controlling Fertility and Bone Turnover

**DOI:** 10.3390/ijms23031633

**Published:** 2022-01-31

**Authors:** Anna Calik-Ksepka, Monika Stradczuk, Karolina Czarnecka, Monika Grymowicz, Roman Smolarczyk

**Affiliations:** 1 Department of Gynaecological Endocrinology, Medical University of Warsaw, Karowa 2, 00-315 Warsaw, Poland; monika.grymowicz@wp.pl (M.G.); rsmolarczyk@poczta.onet.pl (R.S.); 2 Student’s Academic Association, Department of Gynecological Endocrinology, Faculty of Medicine, Medical University of Warsaw, Żwirki i Wigury 61, 02-091 Warsaw, Poland; stradczukmonika@gmail.com (M.S.); karolina.czarnecka.98@wp.pl (K.C.)

**Keywords:** lactational amenorrhea, kisspeptin, lactational amenorrhea method, lactational osteoporosis

## Abstract

Lactation is a physiological state of hyperprolactinemia and associated amenorrhea. Despite the fact that exact mechanisms standing behind the hypothalamus–pituitary–ovarian axis during lactation are still not clear, a general overview of events leading to amenorrhea may be suggested. Suckling remains the most important stimulus maintaining suppressive effect on ovaries after pregnancy. Breastfeeding is accompanied by high levels of prolactin, which remain higher than normal until the frequency and duration of daily suckling decreases and allows normal menstrual function resumption. Hyperprolactinemia induces the suppression of hypothalamic Kiss1 neurons that directly control the pulsatile release of GnRH. Disruption in the pulsatile manner of GnRH secretion results in a strongly decreased frequency of corresponding LH pulses. Inadequate LH secretion and lack of pre-ovulatory surge inhibit the progression of the follicular phase of a menstrual cycle and result in anovulation and amenorrhea. The main consequences of lactational amenorrhea are connected with fertility issues and increased bone turnover. Provided the fulfillment of all the established conditions of its use, the lactational amenorrhea method (LAM) efficiently protects against pregnancy. Because of its accessibility and lack of additional associated costs, LAM might be especially beneficial in low-income, developing countries, where modern contraception is hard to obtain. Breastfeeding alone is not equal to the LAM method, and therefore, it is not enough to successfully protect against conception. That is why LAM promotion should primarily focus on conditions under which its use is safe and effective. More studies on larger study groups should be conducted to determine and confirm the impact of behavioral factors, like suckling parameters, on the LAM efficacy. Lactational bone loss is a physiologic mechanism that enables providing a sufficient amount of calcium to the newborn. Despite the decline in bone mass during breastfeeding, it rebuilds after weaning and is not associated with a postmenopausal decrease in BMD and osteoporosis risk. Therefore, it should be a matter of concern only for lactating women with additional risk factors or with low BMD before pregnancy. The review summarizes the effect that breastfeeding exerts on the hypothalamus–pituitary axis as well as fertility and bone turnover aspects of lactational amenorrhea. We discuss the possibility of the use of lactation as contraception, along with this method’s prevalence, efficacy, and influencing factors. We also review the literature on the topic of lactational bone loss: its mechanism, severity, and persistence throughout life.

## 1. Introduction

According to both WHO and American Academy of Pediatrics recommendations [1,2], every newborn should be breastfed within 1 h of life. Exclusive breastfeeding should continue until the baby is 6 months old. At that age, first complementary foods can be introduced, but breastfeeding continuation is advised up to 2 years of age or beyond. Breastfeeding is associated with various short-term and long-term health benefits for both babies and mothers. Current meta-analysis shows that longer breastfed children are better protected against child infectious diseases and have fewer dental malocclusions and higher intelligence compared to those who are breastfed for shorter periods or not breastfed at all [3]. Moreover, breastfeeding reduces mortality from sudden infant death syndrome and necrotizing enterocolitis [4]. There is also growing evidence that breastfed children may be less likely to suffer from obesity and diabetes in the future as compared to non-breastfed babies [3].

It was proved that breastfeeding improves parental sleep duration and has a positive impact on mood and reaction to stress among breastfeeding women, diminishing the chances of postpartum depression development [5,6,7]. Furthermore, it decreases the risk of breast and ovarian cancers by 26% and 37%, respectively [8]. The risk of type 2 diabetes is also significantly lowered in lactating women [9]. Satisfaction and continuation of breastfeeding depend on many aspects such as time of delivery, diet consumption, malnutrition, healthy habits, educational level, and employment status [10,11].

Amenorrhea is defined as a pause in menstrual bleedings which last more than 6 months during reproductive years. Lactational amenorrhea occurs during postpartum weeks. It prolongs the intervals between following births and thus is related to family history planning [12].

Our study summarizes the effect that breastfeeding exerts on the hypothalamus–pituitary axis and the whole organism, particularly considering fertility changes. Moreover, we discuss the possibility of the use of lactation as contraception, along with this method’s prevalence, efficacy, and influencing factors. We also review the literature on the topic of lactational bone loss: its mechanism, severity, and persistence throughout life.

## 2. The Physiological Aspects of Lactation

During pregnancy, the mammary gland undergoes a series of structural and functional changes in order to prepare itself for milk production. A key role in glandular tissue development is played by three hormones: estrogen, progesterone, and prolactin. Estrogen promotes extensive lactiferous ducts development, precisely ductal elongation, while progesterone and prolactin stimulate profuse alveolar–lobular formation [13]. Estrogen also stimulates prolactin secretion and increases the number of prolactin receptors in mammary gland cells [14]. The action of prolactin in the mammary gland is mediated through its receptor, which activates the JAK/STAT signaling pathway for transcription of genes essential in milk synthesis, e.g., casein [15]. However, during pregnancy, high levels of progesterone block the stimulating effect of estrogen on prolactin, consequently inhibiting milk synthesis until the time of delivery when the placenta is removed [16]. 

The actual process of breastfeeding is controlled by the let-down reflex [17,18]. When a baby is suckling a nipple, it stimulates mechanoreceptors located there. Newly created ascending sensory information is transported all the way up via the spinal cord to reach the hypothalamic paraventricular nucleus (PVN) and stimulate oxytocin production [19]. Oxytocin promotes the contractile activity of myoepithelial cells that surround alveoli. Moreover, oxytocin is the main prolactin-releasing hormone in contrast to dopamine that inhibits prolactin secretion [15]. Prolactin stimulates epithelial alveolar cells to produce milk for the purpose of keeping pace with the baby’s needs [20]. Another neurotransmitter, serotonin, is suggested to be an additional feedback inhibitor of lactation, decreasing milk volume produced [21].

Metabolism-related hormones are also essential in the process of both milk production and mammary gland development. 

Growth hormone (GH), along with prolactin, is essential for milk secretion [15]. It was shown that on the sixth day of lactation, GH on its own can maintain 50% milk production in the absence of PRL, whereas suppression of both GH and PRL can totally eliminate milk secretion [22]. Because of its ability to increase milk volume, growth hormone administration has been used to enhance milk production in women [23]. GH also plays an important role in ductal branching [13].

Insulin, together with glucocorticoids, is essential in the structural mammary gland development by regulating tight junction formation [24] and stimulation of cells differentiation [25]. It was also shown that together with prolactin, they play a key role in the regulation of milk protein synthesis [26].

Suckling provides supply in the breast milk and at the same time ensures lactational amenorrhea. Milk is known as the best food for infants. It provides all necessary compounds for a rapidly growing organism. One of the important components is calcium; its adequate amount is supplied mostly by maternal bone turnover. During lactation, bone remodeling is under hormonal stimulation and can affect lactational osteoporosis due to osteoclasts activation. The longer suckling and lactation lasts, the bigger bone loss occurs. This is further discussed in Section 5.

There are also factors that, at first thought, may not be considered as important in the context of lactation, but in fact, they may cause a major disruption of this process. Such conditions include obesity, alcohol consumption, and stress [16,27,28,29,30,31,32,33,34].

Mother’s well-being is crucial for proper breastfeeding, as it was shown that psychological stress or pain decreases milk volume [35]. It is assumed to be the result of decreased oxytocin release in response to unfavorable conditions. Malnutrition is a factor that stands for smaller milk production and less nutritious milk composition [36].

It is estimated that 82% of women who give preterm birth demonstrate delayed secretory activation. It may also happen in case of delivery by cesarean section, maternal obesity, diabetes or gestational diabetes, and primiparity. Delayed secretory activation is associated with an increased risk of early cessation of lactation [37]. In case of insufficient milk production, called lactational insufficiency, hormonal changes may lead to ovulatory cycles and menstrual bleedings, which increases the risk of short intervals between pregnancies.

Mostly, non-pharmacological strategies, including breastfeeding counseling, regular milk removal, dealing with co-morbidities like anemia, diabetes, hypothyroidism, or quitting smoking, are used in such situations. If this strategy is not efficient, substances increasing milk supply, called galactagogues, may provide support. Galactagogues act mainly by stimulation of prolactin and oxytocin. Pharmacological strategy is based on dopamine antagonists—domperidone and metoclopramide—with proven effectiveness in improving milk production. There are limited data available about the use of sulpiride, growth hormone, recombinant human prolactin, thyrotrophin-releasing hormone, oxytocin, or metformin in case of lactational insufficiency [37]. Group of herbal/dietary galactagogues of unsure effectiveness is widely represented by fenugreek, blessed thistle, fennel, milk thistle, ginger, brewer’s yeast, lactation cookies, or a combination of herbs. In a study by McBride et al., 60% of Australian women tried some method of lactation support with the highest efficacy while using domperidone [38]. Despite the fact that there are no data about the role of galactagogues in returning to regular bleedings, it can be assumed that by the increase in breast-milk supply, we prolong lactational amenorrhea. More research is needed in this field.

## 3. Endocrine Control of Lactational Amenorrhea

The female hypothalamus–pituitary–ovarian (HPO) axis is significantly altered during both pregnancy and lactation [39]. During the pregnancy, placental steroids inhibit gonadotropin-secreting pituitary function, which is reflected by measuring at term LH pituitary content corresponding to 1% of its normal value [40]. After this period, the sucking-induced disruption of the HPO axis leads to the physiologically appropriate amenorrhea. 

Follicle-stimulating hormone (FSH) reaches levels characteristic for the early follicular phase in 4 weeks postpartum, whether or not breastfeeding occurs [41]. The effect of FSH action on follicle development during lactation was observed during an ultrasound exam, which revealed that after 12 weeks postpartum, follicles can reach even 20 mm diameter [42]. It is important to emphasize the fact that these pre-ovulatory-sized follicles showed no or minimal steroidogenic activity due to the inadequate pulsatile LH secretion. Almost throughout the entire period of amenorrhea, until normal menstrual function resumes, the levels of both main FSH-inhibiting hormones (estradiol and inhibin B) remain low, allowing FSH to maintain its concentration in a range typical for the follicular phase [43].

LH plasma concertation may also reach low normal levels within 4 weeks postpartum. However, the disruption in the pulsatile rhythm of LH release seems to block the key events of the menstrual cycle [44]. During the follicular phase of the normal menstrual cycle, the frequency of stimulation reaches 1 LH pulse per hour, whereas, in the study including lactating women, the mean number of significant LH pulses over the 24 h at 4 and 8 weeks postpartum was, respectively, 0.56 and 3.36 [45]. As lactation progresses and the duration of daily breastfeeding declines, the frequency of LH secretion increases to reach frequency characteristic for the normal follicular phase, and this is the first step for fertility resumption [41]. It has been shown that breastfeeding at least five times a day with a duration maintained above 65 min maintains the state of amenorrhea [46]. However, the exact mechanism explaining how the frequency and duration of suckling lead to the suppression of LH pulses is still not exactly explained.

Secretion of both gonadotropins is controlled by gonadotropin-releasing hormone (GnRH), produced and released from GnRH neurons located in the hypothalamus [47]. It is well known that each LH pulse is produced by a corresponding GnRH pulsatile secretion, which means that during the normal follicular phase, GnRH is released once per hour [48]. The release of FSH occurs much less often than GnRH pulses, and low GnRH pulse frequency tends to favor FSH production [49].

According to current knowledge, neuropeptide kisspeptin (Kiss1) is considered as a primary gatekeeper of the HPO axis action, as it is the most potent stimulator of GnRH secretion yet identified [50,51]. Kisspeptins are encoded by the *Kiss1* gene and act through their receptors encoded by the *Kiss1R* gene, which is expressed in the majority of GnRH neurons [51,52]. Mutation in either of these genes is linked to idiopathic hypothalamic hypogonadism and low amplitude of LH pulses in humans, which underscores the role of kisspeptin in the proper function of the HPO axis [49,53]. In rodents, kisspeptin neurons are located in two regions: the arcuate nucleus (ARC) and the anteroventral periventricular nucleus (AVPV), which are involved in GnRH/LH pulsatile secretion and pre-ovulatory LH surge generation, respectively [54,55]. ARC neurons, in addition to kisspeptin, also synthesize and release two co-neuropeptides—neurokinin B (NKB) and dynorphin (DYN) [56]. Neurons that co-express Kiss1, NKB, and DYN are known as KNDy neurons, and current evidence strongly suggests that KNDy cells, as well as KNDy neuropeptides, play an important role in the control of fertility by influencing GnRH activity by acting on their cell bodies and their secretory terminals [57].

The suppression of Kiss1 during lactation may be the key factor in the suppression of GnRH [56]. During lactation in the rat, *Kiss 1* mRNA expression is greatly reduced both in the ARC and AVPV [58]. Furthermore, in the ARC KNDy cells, NKB mRNA is also significantly inhibited [59]. In addition, central administration of Kiss1 during lactation results in increased LH secretion [58], whereas microinjections of selective kisspeptin antagonists into the ARC profoundly suppress pulsatile LH secretion in rats [60].

The reduction in Kiss1 during amenorrhea is suggested to be linked to the occurring in the physical lactation state of hyperprolactinemia [61,62]. A study by Kotani et al. reveals that plasma Kiss1 levels are not totally reduced in lactational amenorrhea, which may suggest that the role of the serum kisspeptin differs from those performed in the brain [63].

**Figure 1:**
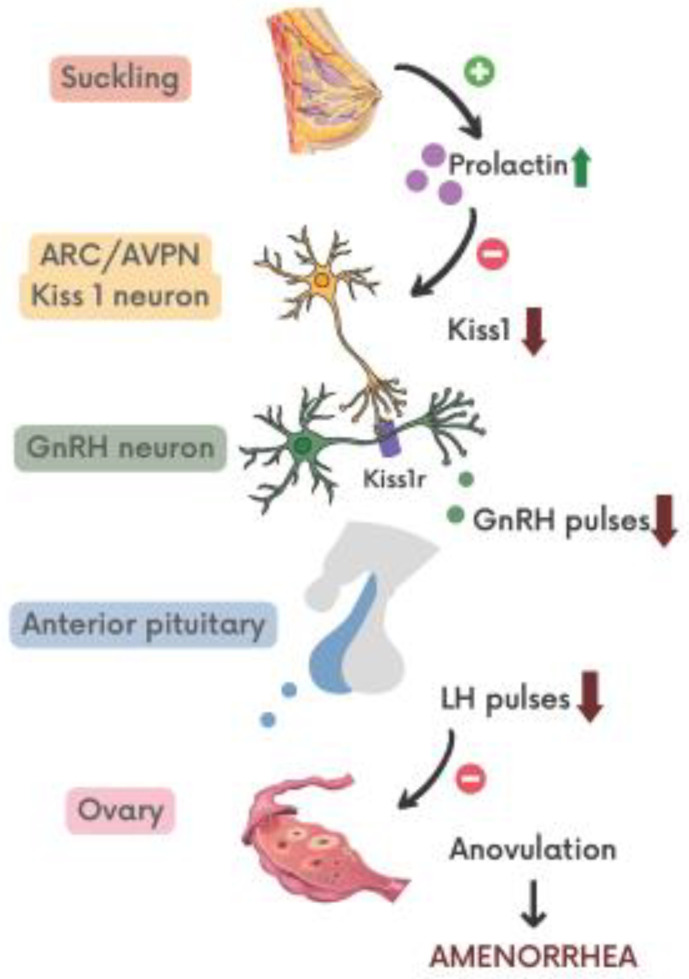
Neuroendocrine control of amenorrhea. ARC—arcuate nucleus; AVPN—anteroventral periventricular nucleus; Kiss1—kisspeptin; Kiss1r—kisspeptin receptor; GnRH—gonadotropin-releasing hormone; LH—luteinizing hormone.

It was shown that infusion of prolactin in female mice suppressed ovarian function by inhibition of GnRH and *Kiss1* mRNA expression, while kisspeptin administration restored normal ovarian function [64]. Research on rats has also shown that PRL acts on ARC neurons to inhibit kisspeptin expression [65,66]. Presented data advocate the role of Kiss1 neurons in the mediation of prolactin’s inhibitory effect on GnRH release (Figure 1).

Even though exact mechanisms standing behind hypothalamus–pituitary–ovarian axis alternation during lactation are still not clear, a general overview of events leading to amenorrhea may be suggested. Suckling remains the most important stimulus maintaining suppressive effect on ovaries after pregnancy. Breastfeeding is accompanied by high levels of prolactin that remain higher than normal until the frequency and duration of daily suckling will be this low to let normal menstrual function resume. Hyperprolactinemia induces the suppression of hypothalamic Kiss1 neurons that directly control the pulsatile release of GnRH. Disruption in the pulsatile manner of GnRH secretion results in a strongly decreased frequency of corresponding LH pulses. Inadequate LH secretion and lack of pre-ovulatory surge inhibit the progression of the follicular phase of a menstrual cycle resulting in anovulation and amenorrhea [67].

## 4. Lactational Amenorrhea as Natural Contraceptive Method

The lactational amenorrhea method (LAM) is one of the family planning methods based on the natural protection of breastfeeding against pregnancy [68].

It is commonly known that during the lactation period, there is a decline in the woman’s fertility, but the exact conditions must be fulfilled to fully exploit the potential of breastfeeding as a method of contraception [69]. 

These conditions were first introduced during the Bellagio Consensus Conference in Italy in 1988 when an international group of scientists gathered to establish the safe and effective use of the lactational amenorrhea method:

1. A period of sixth months after delivery;
2. “Full” or “nearly full” breastfeeding;
3. Postpartum amenorrhea [70].

In that way, women can achieve 98% protection against pregnancy in the first six postpartum months [71].

When any of the above requirements is no longer met, the risk of conception increases, and therefore, another family planning method should be considered to prevent unwanted pregnancy. If no other contraception form is available, women can eventually benefit from breastfeeding alone to maximize the birth interval [71].

The effectiveness of LAM was thoroughly examined and confirmed in a number of papers. One of the earliest studies, conducted even before the Bellagio Consensus Conference by Perez et al., clearly illustrated the relationship between the intensity and duration of breastfeeding and the time of ovulation return [71]. Fewer breastfeeds are significantly connected with ovulating before six months postpartum [72,73], and a similar association is seen in the menses return [72,74]. Some data suggest infant sex correlation with the time of menstrual bleedings’ return with the longer duration with males, mostly seen in malnourished, low energy budget societies. Sons seem to be costlier than daughters [75], although more research is needed to explain this issue.

The lowest ovulation risk and, consequently, the pregnancy rate was obtained during the first six postpartum months [73]. Therefore, this period was included in the recommendations. 

When first menstruation appears during the first six months after the delivery, it is more likely to be anovular than occurring after that time [72]. The chances of ovulation during that time are from 20% to 45%, according to different studies [68,73,76]. Furthermore, the percentage of normal, physiologic ovulations rises from 47% during the first six months to 76% after [73]. The six-month time point is also associated with the appearance of the baby’s first tooth and, therefore, is often connected with supplementary feeding implementation [77].

The term “full or nearly full breastfeeding” means that the baby is given only breastmilk without any other solid or liquid food, or eventually some vitamins, water, juice, or ritualistic feeds in a sporadic way. The introduction of supplementary food causes an abrupt decline in suckling frequency and duration, increasing the risk of ovulation return [78]. During the first six months, the ovulation risk of not menstruating women lowers from about 10% when partial to 1–5% with exclusive breastfeeding [73,76]. However, some studies proved the high efficacy of LAM after excluding the “exclusive breastfeeding” criterium and stated that in this way, the method would be available for more women [79]. 

The pregnancy risk increases significantly with the menses return [80], so to avoid unwanted pregnancy, it is essential to immediately implement another form of contraception [70,79]. Even though first menstrual bleeding is not equal to the complete recovery of fertility, it still gives some information about ovarian activity [81]. In countries where contraception is more difficult to get, it is better to use this amenorrheal period to plan and arrange another contraceptive method than to wait first for the end of the amenorrhea [68,82]. 

Nonhormonal methods of contraception are the best choice while breastfeeding. Women may also consider permanent methods [83,84]. Hormonal methods are possible, but their effect and breastfeeding patterns are still under examination [83]. Research shows that after using LAM, a high percentage of women switch to another method of family planning [85]. The fact that most women tend to resume their sexual activity between 6 and 12 weeks after the delivery only confirms the significance of choosing the most effective contraception method [76,86,87].

Despite the initially established criteria, further research on this topic introduced a couple of factors that might contribute to LAM effectiveness. 

Suckling characteristics such as duration and frequency are the most influential factors, and many studies confirmed their significance [72,73,78]. The minimum suckling frequency needed to suppress ovarian activity is at least five times per day, with a duration of no less than 10 min per feeding. Overall daily suckling time should be not less than 65 min. When the parameters are below these values, there is a higher probability of the ovulational cycle occurring [46]. According to Andersen et al., a frequency of from six to seven feedings is sufficient to maintain anovulation while using supplementary feeding less than once per day [46]. Campbell et al.’s study noticed the importance of median feedings interval [72], which might be directly related to the need for night feed maintenance [78]. Among the supplementary feedings, only the number of bottle feedings was directly related to the frequency of breastfeeding, therefore contributing to the higher risk of ovulation [72].

The way how suckling is discontinued also appears important. Rapid cessation is more likely to be connected with a fertile luteal phase than more gradual weaning [46]. It is presumed that the time of supplementation introduction into the feeding schedule has little influence on LAM efficacy. The level of maternal nutrition also seems to be less significant than suckling behavior [46]. The separation from the baby has a negative impact on LAM. Therefore, the method might not be suitable for working women [88].

Another matter of extreme importance is the acceptance and correctness of the LAM usage among breastfeeding women. Many scientific studies focused on that problem were conducted, and their results vary in different populations (Table 1).

The results express the need to provide more correct information about natural family planning, including the LAM method during contraception counseling [92]. Among some populations, one consultation is not enough, and women need to be reminded of LAM efficacy and advantages throughout the postpartum period [74]. There are societies in which partners tend not to approve of any kind of family planning. In this case, women’s education might be more beneficial if conducted without men’s presence [93]. In India, lactational amenorrhea tends to be a reason not to use any other contraceptive method for 8% of women [94]. Applying effective anticonception in the postpartum period is of extreme importance to both mother and child. According to the World Health Organization, the suggested time before the next pregnancy is at least 24 months [96]. The Conde-Agudelo et al.’s retrospective cross-sectional study shows that when pregnancy occurs earlier than six months after the previous one, there is a higher risk of maternal death, bleeding in the third semester, premature membrane rupture, puerperal endometriosis, and anemia [97]. 

To summarize, lactational amenorrhea method might be effectively used as a contraception method, but only among populations with adequate access to health services or another source of reliable and correct information of its use. Strict adherence to the established criteria increases the safety and reliability of LAM and protects against detrimental health consequences of a too short inter-birth interval.

## 5. The Lactational Bone Loss

Another important process occurring during lactational amenorrhea is the change in bone mineral density (BMD). Pregnancy and lactation bring changes in bone turnover because of the need for building material for the development of the fetal skeleton. There is a significant calcium loss during the lactation period with the breastmilk of 200 mg/day on average, but it can vary even among exclusively breastfeeding women [98]. WHO recommends an extra dose of dietary calcium supplementation during pregnancy.

After the delivery, the rapid change in hormonal status takes place. Induction of lactation is associated with the decline of estrogen and progesterone levels [99]. According to Hillman et al.’s study, the protective effect of estrogens on bones is reduced, and the subsequent lactation period cause increased bone remodeling [99]. The lack of estrogens is believed to be one of the main causes of lactational bone loss [100]. During the late lactation period and after the discontinuation of breastfeeding, estrogens return [99] and cause the formation of the bones [101]. The state of elevated prolactin level, which occurs during lactation, is also associated with the reduced bone mineral content [102]. 

Bone remodeling involves perforating of trabeculae, a decrease in their number, and an increase in cortical porosity. Physical changes occurring during pregnancy and lactation in women such as weight gain, lack of physical activity, and excessive lumbar lordosis play a role in bone and joint loading. In the aim to provide calcium requirements to the fetus or a newborn, the mother’s organism must adapt. During pregnancy, calcium demands are mostly gathered by increased intestinal absorption, while during lactation, calcium homeostasis is gained by increased calcium resorption from bones as a result of the stimulation of osteoclastogenesis and osteoclast-dependent bone turnover [103]. Osteocytes remodel their perilacunar space, which results in enlargement of lacunae and resorption of the perilacunar matrix. Those changes lead to calcium mobilization from the skeleton [104] and temporary bone mineral loss [105].

Due to the lack of estrogen and increased calcium resorption from bones, prolonged lactational amenorrhea can be a potentially threatening condition. Although pregnancy- and lactation-induced osteoporosis (PLO) is a rare disease, in specific conditions like rheumatic diseases, glucocorticosteroids, or oncology treatments, osteoporosis with bone fractures may occur [103].

A couple of factors influence skeleton demineralization during the lactation period. The most important regulators of calcium metabolism in mammals are parathormone (PTH), parathyroid hormone-related protein (PTHrP), vitamin D metabolites, and prolactin. They increase serum calcium levels mostly by bone resorption. Although PTH is one of the most important hormones regulating calcium metabolism in rodents during lactation, its levels were found to be lower [101] or normal [99] in breastfeeding women.

It was proved that mammary cells during lactation produce circulating PTHrP, which might contribute to skeleton resorption [106]. PTHrP suppresses and replaces PTH and, in a lack of estrogen condition, stimulates osteoclasts activity. 

1,25(OH)_2_D (Calcitriol) level was found to be within normal limits in breastfeeding women [99]. During pregnancy, calcitriol’s levels are elevated, stimulating increased calcium intestinal absorption and decreasing its urinary waste. Calcitriol remains elevated even after pregnancy in rodents, while it drops after delivery in humans. Both PTH and calcitriol levels rise during the postweaning period compared to lactation [101]. 

Calcitonin seems to play a protective role during lactation for mothers’ skeleton [105].

To summarize, the main factors that cause bones demineralization during lactation in women are PTHrP secreted by mammary tissue and low estrogens levels, while PTH and calcitriol seem not to play a significant role in this process [105].

Cathepsin K (Ctsk) is a cysteine protease produced by osteoclasts. It is a primary enzyme mediating the degradation of the demineralized bone matrix. Osteocytes’ expression of *Ctsk* genes is elevated during lactation. Ctsk regulates osteocytes expression affecting perilacunar resorption and enlargement of osteocyte lacunae. In addition to the induction of osteocytes, Ctsk also stimulates the induction of osteoclasts. A study by Lotinun et al. on a mouse model reveals that deletion of *Ctsk* decreases perilacunar resorption by osteocytes but also decreases induction of osteoclasts number and their role in bone formation, which normally occurs during lactation in mice [104].

During lactation, the loss of bone mineral density is particularly visible at trabecular-rich sites such as lumbar spine bone [101,107,108,109,110,111] and femoral neck [107,108]. On the other hand, no loss in radius during the 6-month lactation period was observed [107,110]. However, when comparing 30–35-year-old women with the only significant difference between them being the mean lactation duration, radial bone mass at ultra-distal and midshaft sites was lower among the women that breastfed for a longer period [111,112]. 

Bone resorption can be measured by dual-energy X-ray absorptiometry (DEXA) with 4% changes in spine bone mineral content seen just after 3 months of breastfeeding [108]. Atkinson et al. measured the mineral loss in the lower femoral shaft and found the average rate of loss in the bone index of 2.2% during 100 days of lactation [113]. According to Sowers et al., at six months postpartum, breastfeeding women had losses in BMD of the lumbar spine and femoral neck compared to initial values. Hayslip et al. compared exclusively breastfeeding women with the formula-feeding ones, and at six months postpartum, the loss of lumbar spine bone mineral content was associated with breastfeeding, while no significant change happened in the second group [110]. Such comparison was also conducted by Affinito et al. In this study, after six months, both lumbar and radial BMD decreased, again with no significant change in a group of women with bromocriptine inhibited lactation [109] (Table 2).

It seems that the duration of breastfeeding, the volume of produced breastmilk, and the time of returning to regular menses may relate to the magnitude of the changes in bone mineralization. The loss in bone mass was proved to be independent of calcium intake, weight change [107,108], breast-milk calcium concentration, vitamin D-receptor genotype, or use of the progesterone-only contraceptive pill [108]. Therefore, hormonal variations in the postpartum period might be the main trigger of skeleton changes [107].

An interesting observation is that BMD recovery occurs after the cessation of lactation [107,109]. Lamke et al. proved that mothers whose breastfeeding period was shorter than three months first lost and then regained bone mineral content, but there were no losses among those breastfeeding longer [115]. Brembeck et al. showed no decline in bone density if feeding was shorter than 4 months. Longitudinal studies demonstrated an increase in areal bone mineral density (aBMD) seen after cessation of lactation independently of the duration of breastfeeding. However, the same study reveals a decrease in volumetric bone mineral density (vBMD) depending on the duration of feeding, being still important 18 months postpartum for women feeding longer than 9 months [116]. In another study by Bjørnerem et al., lactational changes were irreversible, despite follow-up of 2.6 years post breastfeeding and 3 years post lactational amenorrhea. Residual deficits remained relative to the beginning of breastfeeding with higher cortical porosity, fewer trabeculae, and lower matrix mineralization [117].

A retrospective analysis of 586 postmenopausal women by Yazici et al. showed that lactation length is not an independent risk factor for low femur BMD or low spine BMD, and changes in bone metabolism during lactation had no effect on postmenopausal BMD measured by DXA [118]. Fox et al., in their study, came to a similar conclusion about radius BMD [119]. A prospective observational study among about 6500 women for 16 years showed no associations between parity and duration of feeding with fractures and spine density [120]. Long-term observation reveals possible small, site-specific benefits of parenting and breastfeeding to bone density [121]. The breastfeeding duration of above two years has no significant impact on the postmenopausal fracture risk in most studies [122], although, in one study, the negative association between lactation and hip fracture risk was noticed [123]. 

## 6. Conclusions

Lactation is strictly controlled by a series of reproductive and metabolic hormones that influence both mammary gland development and milk synthesis. Despite the fact that exact mechanisms standing behind hypothalamus–pituitary–ovarian axis alternation during lactation are still not clear, a general overview of events leading to amenorrhea may be suggested. Suckling remains the most important stimulus maintaining suppressive effect on ovaries after pregnancy. Breastfeeding is accompanied by high levels of prolactin that remain higher than normal until the frequency and duration of daily suckling decreases and allows normal menstrual function resumption. Hyperprolactinemia induces the suppression of hypothalamic Kiss1 neurons that directly control the pulsatile release of GnRH. Disruption in the pulsatile manner of GnRH secretion results in a strongly decreased frequency of corresponding LH pulses. Inadequate LH secretion and lack of pre-ovulatory surge inhibits the progression of the follicular phase of a menstrual cycle and results in anovulation and amenorrhea.

The same mechanisms of lactational amenorrhea or anestrus are typical for mammals. Some changes between species may be seen, depending mostly on a suckling or lactating status of an animal, but also factors such as the amount of feeding before and after parturition, level of milk yield, age of the animal, calving difficulty, presence of a bull in the herd, season and its photoperiodism can influence the duration of anestrus in cows and mares [124,125,126]. The nutritional deficit becomes relatively more important during the third- and fourth-week post-parturition [127]. Interestingly, a unique reproductive strategy is represented by swamp wallabies, where females are continuously pregnant and lactating at the same time throughout their reproductive life [128].

If all the established conditions of its use are fulfilled, the lactational amenorrhea method efficiently protects against pregnancy. Because of its accessibility and lack of additional associated costs, LAM might be especially beneficial in low-income, developing countries, where modern contraception is hard to obtain.

LAM is based on the natural and physiologic mechanism of breastfeeding, which allows for its use among religious and ethnic groups. The advantages of breastfeeding for both mother and child and the importance of maintaining the recommended birth interval should be widely spread. Breastfeeding alone is not equal to the LAM method, and therefore, it is not enough to successfully protect against conception. That is why LAM promotion should primarily focus on conditions under which its use is safe and effective. 

More studies on larger study groups should be conducted to determine and confirm the impact of behavioral factors, such as suckling parameters, on the LAM efficacy. 

Lactational bone loss is a physiologic mechanism that enables providing a sufficient amount of calcium to the newborn. Despite the decline in bone mass during breastfeeding, it rebuilds after weaning and is not associated with a postmenopausal decrease in BMD and osteoporosis risk. Therefore, it should be a matter of concern only for lactating women with additional risk factors or with low BMD before pregnancy.

## Figures and Tables

**Table 1:** Summary of LAM acceptance and correctness in selected countries.

Reference	Country of the Observational Study	Duration of Amenorrhea with LAM	Women Amenorrheic after Six Months	Main Results of the Study
[89]	Australia	Median: >8.5 months	-	Breastfeeding is an effective contraception method during the first six months after the delivery.
[90]	Uganda	-	62.7%	It is possible to use LAM as a contraception method for most women. More support from health workers is needed.
[85]	Multicenter study (Egypt, Mexico, Nigeria: Jos, Nigeria: Sagamu, Philippines, Germany/Italy, Sweden, United Kingdom, United States)	-	65.2%	LAM method use is highly satisfactory and effective without extensive supervision.
[74]	Mexico	Mean: 5.5 months (mean duration of LAM use was 4.3 + 0.2 months)	-	In developing countries, LAM use might be improved by regular supervision.
[91]	Turkey	-	56.2% (women who had 6-month-old infants)	Prenatal and postnatal counseling is needed for effective LAM use because of low LAM criteria fulfillment.
[92]	Nigeria	-	-	There is a need for correct information about natural family planning methods.
[93]	Niger	-	-	The improvement of women’s education about LAM criteria and better access to health services is needed.
[94]	Ethiopia	-	-	The low level of knowledge about LAM might be improved with home-to-home counseling.
[95]	Tanzania	-	-	Women do not know about LAM. Future counseling should address their misconceptions, concerns, and knowledge gaps.

Legend: “-“—no data; LAM—lactational amenorrhea method.

**Table 2:** Decrease in BMD depending on the site of measurements and the duration of lactation.

Study by	Time of Observation	Lumbar Spine	Femoral Neck	Radius
Laskey [108]	3 ms	3.96%	2.39%	n/d
Atkinson [113]	100 days	n/d	2.2%	n/d
Sowers [114]	6 ms	5.1%	4.8%	n/d
Hayslip [110]	6 ms	6.5%	n/d	NS
Affinito [109]	3 ms	6%	n/d	2%
	6 ms	7.5%	n/d	5%
Cross [101]	3 ms	4.3%	n/d	+5.7%
Kolthof [107]	3 ms	5.2%	n/d	n/d

Legend: BMD—bone marrow density; ms—months; n/d—no data; NS—no significant differences.
